# Engaging Transformation: Using Seasonal Rounds to Anticipate Climate Change

**DOI:** 10.1007/s10745-021-00269-2

**Published:** 2021-09-29

**Authors:** Karim-Aly Kassam, Morgan Ruelle, Isabell Haag, Umed Bulbulshoev, Daler Kaziev, Leo Louis, Anna Ullmann, Iriel Edwards, Aziz Ali Khan, Antonio Trabucco, Cyrus Samimi

**Affiliations:** 1 Department of Natural Resources and the Environment, Cornell University, Ithaca, NY USA; 2 American Indian and Indigenous Studies Program, Cornell University, Ithaca, NY USA; 3 Department of International Development, Clark University, Community & Environment, Worcester, MA USA; 4 Department of Geography, University of Bayreuth, Bayreuth, Germany; 5 Center of Ecology and Environmental Research, Bayreuth, Germany; 6 School of Professional & Continuing Education, University of Central Asia, Khorog, Tajikistan; 7 Mountains Societies Research Institute, University of Central Asia, Khorog, Tajikistan; 8 Impacts On Agriculture, Forests and Ecosystem Services Division, , CMCC Foundation—Euro-Mediterranean Centre on Climate Change, Sassari, Italy

**Keywords:** Ecological calendars, Indigenous and local knowledge, Dakota/Lakota Oneida Lake, Pamir Mountains

## Abstract

Seasonal rounds are deliberative articulations of a community’s sociocultural relations with their ecological system. The process of visualizing seasonal rounds informs transdisciplinary research. We present a methodological approach for *communities of enquiry* to engage *communities of practice* through context-specific sociocultural and ecological relations driven by seasonal change. We first discuss historical précis of the concept of seasonal rounds that we apply to assess the spatial and temporal communal migrations and then describe current international research among Indigenous and rural communities in North America and Central Asia by the creation of a common vocabulary through mutual respect for multiple ways of knowing, validation of co-generated knowledge, and insights into seasonal change. By investigating the relationship between specific biophysical indicators and livelihoods of local communities, we demonstrate that seasonal rounds are an inclusive and participatory methodology that brings together diverse Indigenous and rural voices to anticipate anthropogenic climate change.

**Supplementary Information:**

The online version contains supplementary material available at 10.1007/s10745-021-00269-2.

## Introduction

To recognize and respond to climate change, we must appreciate the complex connectivity between sociocultural and ecological systems. Relational thinking is fundamental to grasping the implications of climate change at the scale of local communities (Gaston *et al.*, 2018). Developing a relational understanding of climate change confounds disciplinary approaches because it forces us to think across epistemological and ontological boundaries. A being “is” in relation to a continuum of beings within its habitat (Ingold, 2000). Furthermore, there is a “necessary unity” between humanity’s relations to its physical and informational environment (Bateson, 2002). Living organisms, including humans, evolve through relations with their habitat across space and time, corresponding to the rhythms of seasonal change within their respective landscapes (Dunlap *et al.*, 2004). From an evolutionary standpoint, populations of organisms continuously adapt – at different rates – to climate variability (Petit & Hampe, 2006). Similarly, human knowledge derives from engagement and performance in the spatial and temporal dimensions of climatic and ecological cycles. Therefore, insights into seasonal changes and climatic variation emerge from the sociocultural and ecological relations of communities with their habitats (Kassam, 2009).

Engaging the complex connectivity between humans and their habitats is empirically demanding and nuanced. Adaptation to climatic changes requires transformation of ecological, sociocultural, and economic structures in order to be effective (Klein *et al.*, 2014). Investigation of the impacts of climate change at the scale of villages and towns requires not only *interdisciplinary* collaboration among the sciences and humanities, but *transdisciplinary* partnerships with Indigenous or local knowledge holders.^1^ People engaged in farming, fishing, gathering, herding, hunting, tending orchards, and other ecological professions are *communities of practice* who contribute to the cogeneration of knowledge with *communities of enquiry* from the social and biophysical sciences as well as the humanities (Argyris *et al.*, 1985; Kassam, 2009). Henceforth, we use the term *communities of practice* and *enquiry* to describe these diverse constituents to knowledge cogeneration. Based on these conceptualizations of transdisciplinarity and relational thinking, we here provide a participatory methodology for collaborative and applied research to develop anticipatory capacity for climatic change in rural and Indigenous communities (Kassam *et al.*, 2018).^2^

Seasonal variation is fundamental to life on earth. Across the globe, ecosystems rely on biophysical rhythms, i.e., cycles of temperature, precipitation, and the development and behavior of organisms. Human food systems also depend on seasonal change (Kassam, 2021). These cycles are never entirely predictable, and communities across the globe have developed mechanisms to deal with varying degrees of uncertainty. However, rapidly increasing concentrations of greenhouse gases in the atmosphere are driving unprecedented climate changes, including increasing weather variability and extreme events, that pose existential challenges to humanity (Field *et al.*, 2014). Unfortunately, people who contribute least to anthropogenic climate change are facing its harshest impacts to their food systems (Kassam *et al.*, 2011, 2018). Communities of enquiry located in industrialized nations most responsible for climate change are obligated to generate knowledge that has immediate application for adaptation and to build anticipatory capacity for increasing climate variability (Tschakert & Dietrich, 2010). However, such efforts require close collaboration with affected communities throughout the research process, to include their nuanced knowledge of their habitat, co-generate new knowledge to address their priorities, and disrupt the power structures that prevent climate justice (Haverkamp, 2017; Kassam, 2013).

Anthropologists and geographers use the term ‘seasonal round’ in reference to patterns of human activities associated with cyclical changes in their ecosystem. The seasonal round of a community is based on – and thus reflects – knowledge of complex relationships among biological and physical processes as well as sociocultural phenomena (Turner, 2014). Seasonal rounds are often recounted as place-based narratives that give insight into the living world and the sequence of festivals and other events that link a people to their habitat. Seasonal rounds lend themselves to a wide aesthetic, and therefore may be described through texts, tables, graphs, and circular figures illustrating a calendar of events expressing human ecological relations. As one element of participatory research, the discussion and visualization of seasonal rounds enable communities of practice and enquiry to co-generate knowledge that is multidimensional, layered, and nuanced with respect to human-ecological relationality in space and time. The development of seasonal rounds is an ethnographic approach to establish a common vocabulary for dialogue about change and gain insight from local and indigenous knowledge holders. As a participatory methodology, seasonal rounds not only facilitate understanding of the entangled implications of anthropogenic climate change but generate insight as to how communities might develop context-specific adaptation strategies that anticipate and respond to emergent climate challenges.

Specifically, we use seasonal rounds to develop and revitalize ecological calendars that build anticipatory capacity for climate change at the community level. In previous work, we described ecological calendars as: “knowledge systems to measure and give meaning to time based on close observation of one’s habitat.” Comprised of seasonal indicators that include physical phenomena, such as the first snowfall or last frost, as well as biological events, such as the flowering of certain trees or the arrival of a migratory bird species, these calendars do not rely solely on fixed cycles of the sun, moon, or stars. Measurement of time is flexible with respect to celestial cycles, and communities can identify the optimal timing for their livelihood activities. Consequently, ecological calendars may enhance anticipatory capacity for adaptation to anthropogenic climate change because communities synchronize their activities with their ecosystem (Kassam *et al.*, 2018: 250).

Construction of a community’s contemporary seasonal round is essential to develop an *ecological calendar*. A seasonal round has two spatial and temporal components, respectively. The spatial dimension includes: 1) *occupancy* of diverse landscapes by human communities, and 2) seasonally determined patterns of *movement* or *migration* within those landscapes, e.g., agropastoral communities moving livestock across winter, spring, summer, and autumn pastures or the migration of hunting societies through different ecological zones to secure their food supply. The temporal dimension includes: 1) knowledge of *seasonal indicators* that inform the timing of livelihood activities; and 2) *intergenerational transfer* of this cumulative knowledge to secure future livelihoods, e.g., farmers start ploughing their fields based on the snow-cover along mountainsides or the return of spring peepers (*Pseudacris crucifer*). Such knowledge is passed down through the generations.

Our goal is to demonstrate the process of developing seasonal rounds as a participatory research methodology to anchor transdisciplinary climate change research at a community scale rather than to present research findings. We first explore the history of scholarship on seasonal rounds across a wide range of sociocultural and ecological contexts. We then present ongoing transdisciplinary climate change research in North America and Central Asia in which seasonal rounds serve as a starting point for revitalization^3^ and development of ecological calendars, and reflect on how the collaborative process of articulation and visual expression of seasonal rounds can facilitate cogeneration^4^ of actionable insights. We conclude with a synthesis of lessons learned.

## Ethnographic History of the Seasonal Round

It is important to review the work of ethnographers in the disciplines of anthropology, geography, and human ecology who have long been interested in seasonal patterns of life, and how those patterns relate to the local environmental conditions. Franz Boas’s first major ethnographic contribution to cultural anthropology, *The Central Eskimo*, detailed the seasonal relationships of the Inuit of Baffin Island with their habitat in 1883 and 1884 (Boas, 1964). Inuit settlement and migration were directly driven by their perception of biophysical indicators to secure their food through hunting, fishing, gathering, and trade.^5^

Similarly, Boas’s student, Alfred Louis Kroeber acknowledged the role of ecological context and how the relationships human societies have with their habitat contribute to the diversity and geographic distribution of cultures. While sensitive to the dangers of environmental determinism common at this time, Kroeber recognized that cultures were dynamic processes emerging from their relations with the biophysical aspects of their ecology. His *Cultural and Natural Areas of Native North America* (1939) examined the relations of indigenous cultures to seasonal change, including temperature and precipitation patterns as well as engagement with plants and animals. Despite being limited by his colonial perspective, this work outlined the connectivity of human cultures to the biophysical rhythms of their habitat, which in turn influenced their ecological professions,^6^ livelihoods, and food systems. While not directly referring to seasonal rounds, he argued that diverse cultural manifestations emerge from a complex connectivity of relations within and across ecological zones.

Kroeber’s student Julian Steward (1938), a founder of cultural ecology and ecological anthropology, studied the movement of Western Shoshone bands over a year as a model of adaptation to the seasonal availability of plants and animals across the Great Basin to understand the particularities and similarities of cultural development under comparable environmental conditions. Steward combined Boas and Kroeber’s ideas about the context-specificity of culture with an analysis of universally shared patterns of human relations across diverse environments (Orlove, 1980; Thomas, 1973).

Evans-Pritchard (1939-1940) was among the first anthropologists to visualize time as a cycle of interaction between social and ecological phenomena. While Boas, Kroeber, and Steward mapped human sociocultural and ecological relations with their habitat, Evans-Pritchard provided additional insight by illustrating the seasonal cycle as distinct but not exclusive of geography or place. His work with the Nuer in what is now South Sudan documented their reckoning of time as flexible in relation to lunar cycles as well as climatic and ecological processes. His circular diagrams of the Nuer seasonal round elucidated connections between weather and human sociocultural and ecological relations (Evans-Pritchard, 1939: 197–198, 1940: 98–99).

In the late twentieth century ethnographers increasingly investigated the interactions between space and time. Basso (1996) articulates how Western Apache communities integrate spatial and temporal relationality with their landscapes. Turner (2014: vols. 2, 4) describes seasonal rounds as “patterns of seasonal movement and residence … within and across diverse geographic and ecological areas, [which] reflect and embrace the complex systems of knowledge and practice that integrate all the different aspects of peoples’ lifeways.” This movement of people across territories has been deliberate, following a predictable pattern to engage landscapes based on knowledge of seasonal change across space. The relationships between weather, its ecological consequences, and human action are fundamental to survival (Bearchum, 2007; Ridington, 2013; Turner, 2014). While continuity of the seasonal round requires sharing of knowledge across generations, patterns of activity necessarily change over time in response to environmental as well as sociocultural change. For example, colonization and forced sedentarization have had dramatic impacts on the seasonal rounds of many indigenous peoples, which were informed by ecological indicators and livelihood requirements.

Scholars of indigenous ecological knowledge have studied seasonal rounds to explore the mutually reinforcing relationship between culture and habitat. As part of their stewardship practices, diverse cultures have developed rules regarding utilization of plants and animals in time and space (Burch, 1998, 2005; Kassam, 2010, 2015; Lantz & Turner, 2003; Woodward & McTaggart, 2019). Mindful relations with plants and animals include carrying out activities such as gathering or herding at specific times of year to the benefit of those organisms (Turner, 2014). Furthermore, arrangements between cultural groups practicing different ecological professions (e.g., farmers and herders) to occupy the same spaces at different times of year is fundamental to their relations with their habitat and each other (Kassam, 2010).

Earlier work consistently demonstrated the value and depth of indigenous ecological knowledge to address context-specific challenges, but biophysical scientists are increasingly recognizing how such knowledge can inform responses to global problems, including climate change (Alexander *et al.*, 2011; Fernández-Llamazares *et al.*, 2017; Haag *et al.*, 2021; Reyes-García *et al.*, 2016; Ruckelshaus *et al.*, 2020; Woodward *et al.*, 2012). As the potential of indigenous ecological knowledge becomes more widely recognized scientists, collaborations between communities of enquiry and practice require close attention to power dynamics so that the research process is not extractive (Kassam, 2013; Nadasdy, 1999). In many cases, the impetus to illustrate seasonal rounds comes from knowledgeable community members concerned about loss of knowledge for future generations. Woodward (2010) demonstrated the use of seasonal rounds among aboriginal communities in Australia as a way to sustain their relations with their habitat based on respectful and reciprocal partnerships between scientists and indigenous communities (Woodward & McTaggart, 2019; Woodward *et al.*, 2012). The process of generating and visualizing seasonal rounds can be integral to building strong and respectful working relationships within and among these communities.

## Application of Seasonal Rounds to Climate Change Adaptation

**Fig. 1:**
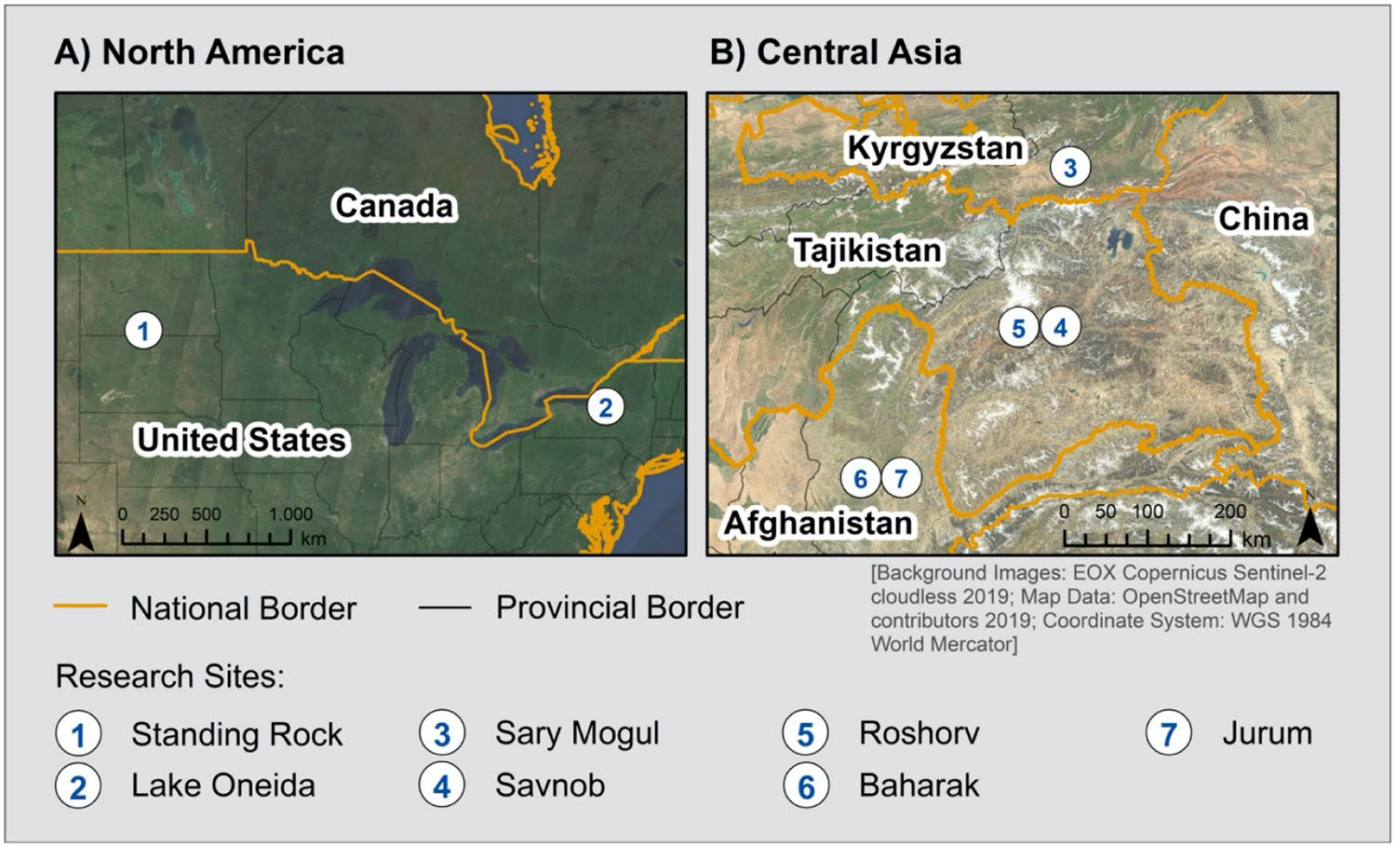
Locations of research sites in North America and Central Asia

**Fig. 2:**
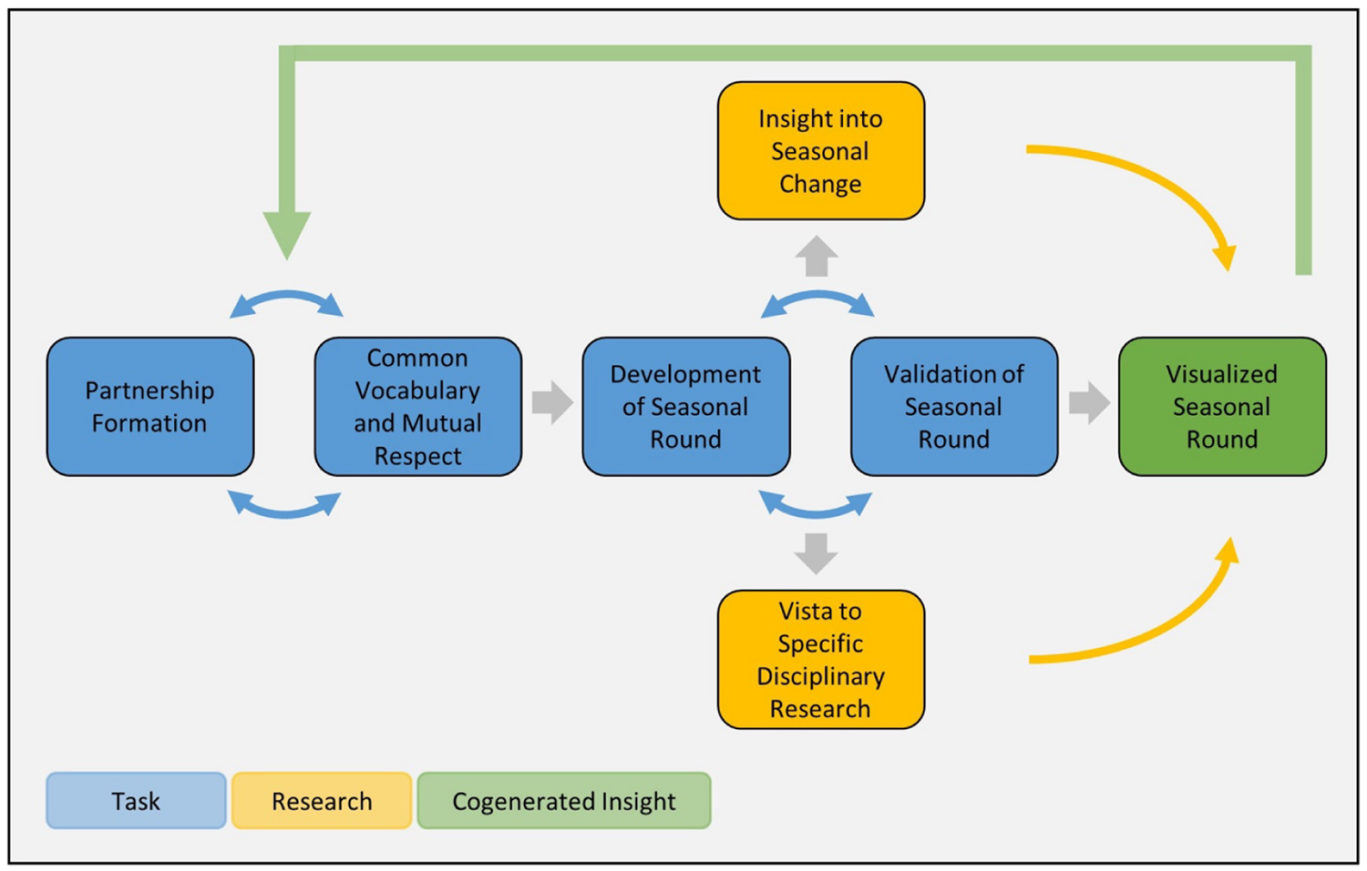
Dynamic process of creating seasonal rounds

Based on the historical development of seasonal rounds and our own recent research (Supplementary Materials 1), we applied this process in a new action research initiative (2015–2021) that would facilitate engagement between communities of practice and enquiry to generate mutual understanding and insights. The project brought together social and biophysical scientists and their students with Indigenous or rural communities in North America and Central Asia (Fig. 1, Table 1; see Supplementary Materials 2 for short descriptions of the study sites). Collaboration in the co-creation of seasonal rounds served as the primary methodology to facilitate communication among participants and generate new insights about the impacts of climate change and possibilities of adaptation to increasing climate variability (Fig. 2, Table 1). Below, we provide a distillation of effective techniques learned through this approach. The process is simultaneously an act of participation and generation of research outcomes.

**Table 1:** Research contexts, including geographical locations and basic demographics

Location	Latitude	Longitude	Elevation (meters asl)	Population	Majority cultures
Standing Rock Nation (USA)	46°05′13″ N	100°37′48″ W	500	8,581	Lakota, Dakota
Oneida Lake (USA)	43°10′22″ N	75°56′12″ W	120	266,000	Euro-American
Sary Mogul (Kyrgyzstan)	39°40′33″ N	72°53′03″ E	2985	5,156	Kyrgyz
Savnob (Tajikistan)	38°19′58″ N	72°24′32″ E	2675	310	Bartangi
Roshorv (Tajikistan)	38°19′00″ N	72°19′20″ E	3040	1,200	Bartangi
Baharak (Afghanistan)	37°00′00″ N	70°53′00″ E	1470	46,093	Tajik, Uzbek
Jurum (Afghanistan)	36°51′50″ N	70°49′50″ E	1560	50,190	Tajik Uzbek

### Partnership Formation

Long-term research relationships facilitate communication and contribute to a growing trust relationship. In each of the research sites except Oneida Lake, USA, members of the research team had established long working relationships, at least 10 years, with the respective community partners. Awareness of and sensitivity to the diverse cultural contexts further made formation of research partnerships possible. The research team usually initiated the partnership with the secular leadership such as the Tribal Chairman or Village Organization President followed by a similar conversation with local spiritual leaders such as *Khalifas*, *Imams*, or Traditional Elders. The conversation with community leaders centered on ecological calendars as a practice with which they could identify both historically and culturally. The project objectives, its collaborative nature, and expected outcomes were also discussed to allow them to assess the potential benefits of this approach for anticipating climatic variation.

**Fig. 3:**
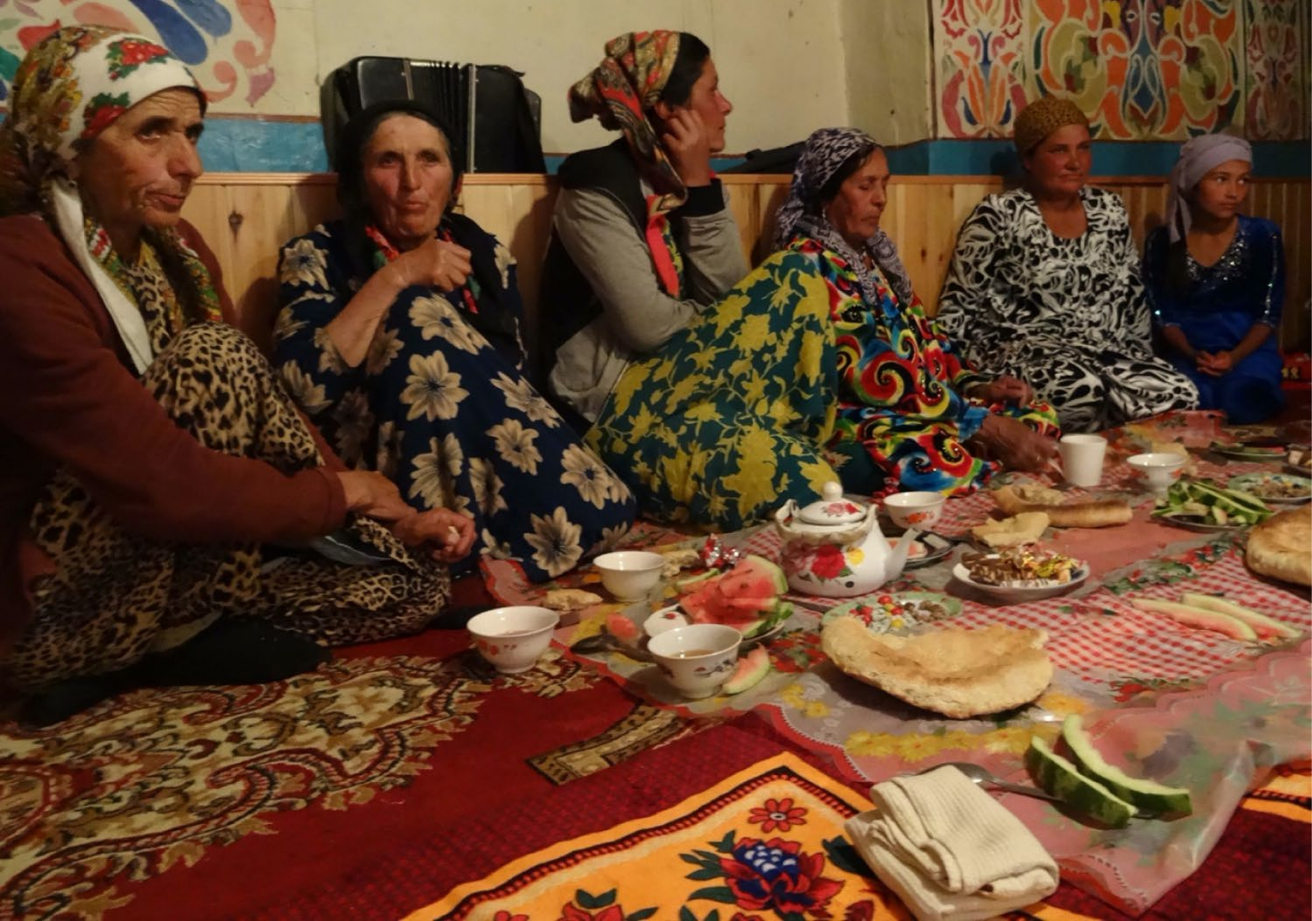
Meal with Community Members in Savnob during the Inception Workshop, 2017. Photo Credit: Isabell Haag

Once there was unanimous agreement to proceed with research, we arranged a formal inception workshop accompanied by a meal (Fig. 3). We identified participants representing various ecological professions who could contribute to the process of knowledge cogeneration. Cultural context determined the means of contact. Whereas in North America, participants were contacted by phone, email and letters, in the Pamir Mountains of Central Asia personal visits were necessary. A community leader or key member of the research team visited community members in their homes or fields. Similarly, in the Standing Rock Nation, it was appropriate to visit Elders to invite them to the workshop.

At Oneida Lake, the only research site consisting of non-indigenous Euro-American settlers, the community of enquiry did not have established relationships. However, since, Cornell University has maintained a Biological Field Station at Shackleton Point, located on the south shore of the lake for over 60 years, staff who live within the watershed assisted in identifying potential knowledge holders. In addition, Cornell Cooperative Extension staff from the four counties within the watershed also suggested individuals.

At the inception workshop, we made a formal audio-visual presentation about the project as the participants gathered for a meal. The community of enquirers*,* including the students, were introduced to the community of practice. The funding agencies and contributing institutions were identified. The objectives of the project were explained and the need for anticipatory capacity for climate change was discussed, linking it specifically to the community’s food and livelihood systems. We described the geographic diversity and locations of all research sites to demonstrate the breadth of the project (Supplementary Materials 2), introduced the notion of ecological calendars and described the connections to the respective community’s ecological context. Finally, the expected outcomes and products resulting from the project were explicitly discussed. Informed consent for participation was obtained. The meal was prepared by a community member but hosted and financed by the community of enquirers. The principal investigators and students served the participants. The meal began with an invocation of thanks usually led by a locally recognized spiritual leader.

In addition to providing the basis for building trust relationships between the community of practice and enquirers, the meal itself was an impetus for a conversation on achievements of local food sovereignty as a key outcome of the project. While the meal was hosted by the community of enquirers, the community of practice also tended to the needs of the hosting-visitors. This event provided an opportunity to learn about their diverse backgrounds, discuss the project and its potential relevance, and tend to one another. The meal was the first concrete event where mutual care and respect were established.

While women were present, their numbers varied according to cultural context, especially in Central Asia (Table 2). For example, in the religiously conservative community of Sary Mogul, trust needed to be established and consent provided by the *Aksakals* (a Kyrgyz term for male Elders) before researchers could speak with both women and men. Other sociocultural factors led to women participating more in individual interviews than in public events such as the inception and validation workshops. First, the transdisciplinary research group tended to arrive in large teams of scientists and their students, and women were reticent to engage such a large group of outsiders. Second, a few of the women in the villages of Savnob and Roshorv did not consider themselves experts about the local landscape because they were from other villages and had married into the community. Nonetheless, men openly acknowledged the skills of those women, identifying one as a skilled hunter and another as the most knowledgeable orchardist. Third, during the summer field season, the burden of labor on women is high. Women have less time to participate in research due to agricultural as well as domestic responsibilities. The best time to talk to women would be in late autumn, winter, and early spring, but these villages are not accessible at these times because of inclement weather and poor road conditions, and the constraints of the academic calendar. We explicitly acknowledge low participation of women as a weakness. Potentially, graduate students have an opportunity to spend the whole calendar year in communities to access women’s insights and experiences. As the research project is iterative, women’s embodied knowledge needs to inform future research. To ignore women’s knowledge would be perilous for effective transdisciplinary research.

**Table 2:** Locations and dates of workshops and other research activities to develop seasonal rounds for climate adaptation

Location	Inception Workshop	Further research activities	Validation Workshops
Standing Rock Nation (USA)	December 8–10, 2015: 34 participants (20 females, 14 males)	8 community meetings, 43 participants (26 females, 17 males); 8 individual interviews, 10 participants (6 females, 4 males)*	October 8–11, 2018: 32 participants (30 females, 12 males)
Oneida Lake (USA)	June 3–4, 2016: 20 participants (6 females, 14 males)	45 individual interviews, 55 participants (15 females, 40 males)*	February 22–23, 2019: 18 participants (6 females, 12 males)
Sary Mogul (Kyrgyzstan)	July 13, 2016: 24 participants (all male)	39 individual interviews, 39 participants (20 females, 19 males)	July 12, 2018: 25 participants (3 females, 22 males)
Savnob (Tajikistan)	June 20, 2017: 20 participants (5 females, 15 males)	20 individual interviews, 20 participants (6 females, 14 males	July 2, 2018: 23 participants (4 females, 19 males)
Roshorv (Tajikistan)	June 27, 2017: 18 participants (3 females, 15 males)	17 individual interviews, 17 participants (3 females, 14 males)	July 6, 2018: 19 participants (3 females, 16 males)
Baharak (Afghanistan)	June 27, 2018: 14 participants (all male)	NA	NA
Jurum (Afghanistan)	June 28, 2018: 14 participants (all male)	NA	NA

^*^Including participating spouses, parents and friends

### Creating a Common Vocabulary and Mutual Respect for Different Ways of Knowing

Developing seasonal rounds simultaneously requires and establishes a common vocabulary. It also stimulates reciprocal respect for different ways of knowing between and among scientists and local knowledge holders. As the research was undertaken by a multidisciplinary team working across different conceptual paradigms, mutual understanding and respect need to grow from the beginning of the research process. Therefore, to create an enabling environment for cogeneration of knowledge, the valorization of different ways of knowing first had to occur among the members of the community of enquiry for each other; and second, appreciation of the depth, breadth, and diversity of Indigenous or place-based knowledge among the heterogeneous members of the community of practice followed.

Documentation of seasonal rounds is a means of focusing the community of enquiry by providing a common basis to begin their research. During this process, relevant terminologies have to be clarified to ensure mutual understanding between the research team and the local knowledge holders. For example, identifying culturally specific starting points of seasons or how communities understand the notion of “rain”, required conversation. While conducting interviews on a rainy day in Savnob, a rare occurrence in the summer in the Western Pamirs, the research team discovered different understandings of the word “rain”. Although it was raining outside, one of the villagers said that they do not experience rain in the summer. Through discussion the scientists realized that they were using a meteorological definition of rain, which would include any kind of liquid precipitation, while the community members differentiated *tsirak pirak*, an onomatopoeia for soft rain that does not wet the earth or destroy crops, from *sharrast thyad*, another onomatopoeia for harder rain that soaks the ground and can damage crops. As it is *sharrast thyad* that matters to local livelihoods, villagers were only considering the heavier rain when answering questions.

Creating a shared understanding and mutual respect cannot be achieved within one field season. The reoccurrence of research steps required by the effective development of seasonal rounds, including iterative stages of data gathering and validation, provides continuity for the research process. It also sustains long-term relations between the community of enquiry and community of practice. It is through this process that members of the community of practice also began to recognize the heterogeneity of knowledge and experiences among themselves. This creates further opportunities for collaboration and common understanding, so that the research project gains meaning and relevance for all involved.

### Development of Seasonal Rounds

As a fundamental component of partnership formation and building trust, the inception workshops were designed to establish at the outset the collaborative process of knowledge generation which is a key tenet of this transdisciplinary research initiative. Therefore, the idea of seasonal rounds was also introduced and collectively visualized. Creation of seasonal rounds at workshops enabled conversation of the phenomenological reality faced by the community of practice with respect to seasonal change, climatic variation, and their respective impacts on the local livelihood and food systems.

The process of discussing and illustrating a seasonal round begins with a facilitator from the community of enquiry explaining the purpose of the activity and explaining how the activity will proceed. The research team places a large sheet of paper printed with a series of concentric circles on the wall. The first questions pertain to the seasons, for example: How do you know the winter has ended? How do you know the next season has begun? How many seasons follow? What are the names of those seasons? The answers to these questions are used to identify important reference points on the seasonal round to which participants can relate other knowledge that emerges through discussion.

Discussion of the seasonal round starts at a specific time of year and proceeds through the seasons. In Standing Rock, the annual cycle begins with the first singing of the Western meadowlark (*tȟašíyagmuŋka*, *Sturnella neglecta*), so discussion began with spring and proceeded through subsequent seasons. At Oneida Lake, the conversation started with seasonal changes occurring at the time of the workshop and then moved through the rest of the year. The discussion of processes and events often reminds participants of related phenomena and human activities in seasons that have already been considered, and it is important to document such knowledge whenever it arises. Thus, from the very beginning, developing seasonal rounds is an iterative process, rather than a strictly linear one (Fig. 2).

As the discussion moves from season to season, the facilitator gears questions toward the specific ecological professions within the community. For example, the facilitator might ask herders questions about the time for moving animals, whereas farmers will know more about the best time to plant and harvest each crop, and other relevant agronomic activities. Other knowledge, such as the timing of sociocultural events like festivals and celebrations, are typically shared across the community. Furthermore, one of the roles of the facilitators is to encourage participants to share what may seem to be contradictory knowledge based on different experiences. Engaging this diversity of knowledge is often challenging, especially when some participants are regarded as authorities. Facilitators emphasize that the goal is not to achieve consensus, but to enrich the seasonal round with knowledge derived from a diversity of experience.

Concerns about climate change impacts often arise on their own, but it is important to ask questions about these impacts once most of the seasonal round has been documented. The iterative process allows for emergence and discussion of more observations, concerns, and insights. Therefore, the facilitator asks if participants have noticed any changes in the weather in the past 10 years. Participants often describe the uncertainty and anxiety associated with climate change, particularly the increasing frequency of unusual and extreme weather events. These discussions inform the community of enquiry as to the need for further research that will enhance the anticipatory capacity of the community of practice. In addition to climate change, participants often raise immediate priorities such as access to education, malnutrition, and poverty. The fact that such concerns arise indicates that climate change is already exacerbating existing inequities.

During the process of generating seasonal rounds, members of the research team are assigned various tasks. A community researcher assists with both translation and explanation. In many cases, Elders also help with translation and elaboration across various languages. Another team member records information on the circular diagram so that participants can review what is documented. Different colored markers are used to categorize information, e.g., green is associated with plants, brown with animals, and red with hazards. Meanwhile, other members of the community of enquiry listen attentively and take detailed notes to ensure that the nuances of the conversation are captured from different disciplinary perspectives. If culturally appropriate and accepted by the community, the process is photographed, leaving not only a written but also a complementary visual record. After the community meeting, documentation of the seasonal round continues with individual interviews.

### Validation

As information through individual and group interviews is gathered, compiled, and analyzed, an empirically rich and more detailed seasonal round emerges. The community of enquirers returns to the respective research site to share their findings through a validation workshop. Again, discussions are first held with the secular and religious leaders. Then a community wide meeting is called, inviting all the original participating members, individual interviewees, and others who are interested to the validation workshop. As in the inception workshop, a locally prepared meal hosted by the research team provides the social context for discussion and insight. A new seasonal round based on information from interviews that has been gathered and analyzed is presented. The key questions are: Is the seasonal round accurate? Did the community of enquirers understand the community of practice? What was misunderstood? What is missing? Validation workshops were conducted at all research sites between 2018 and 2019 (Table 2).

The process allows for corrections, greater nuance, more information, and new insights. At the validation workshop in the village of Savnob, participants arrived 45 minutes early at the local schoolhouse, keen to get started and ask questions. An important new insight from the workshop was that ploughing depends on the availability of oxen. There are six oxen available in Savnob, and a pair is required for ploughing. Villagers must borrow extra oxen from the village of Roshorv at the start of Spring. Therefore, even if biophysical cues indicate time for ploughing, there is a delay based on availability of oxen. This shows that when designing an *ecological calendar*, other factors such as availability of labor and other resources influence livelihood activities that are informed by seasonal indicators.

At the validation workshop in Roshorv, an important correction to the collected information was that villagers also had agricultural lands in the nearby village of Yapshorv, which is located at a lower altitude. There is at least a 25 to 30-day time lag in key agricultural activities between Yapshorv and Roshorv. Lands from both these villages contributed to the food security of the people of Roshorv, so it was necessary to disaggregate the seasonal indicators that had been collected for these two villages. Furthermore, it became clear during validation that physical hazards may serve as cues for livelihood activities. Increasingly, landslides are a major threat to mountain communities resulting from environmental degradation and then exacerbated by climate change. In fact, villagers explained that a major landslide and subsequent destruction of houses in Yapshorv led to the settlement of Roshorv. Over time, as landslides become part of the seasonal cycle of mountain communities, villagers have developed capacity to anticipate their impact on their livelihood activities. For example, in Yapshorv, farmers associate the first landslide with the ripening of apricots.

In transdisciplinary research, it takes time for a common understanding and insights to emerge. For instance, until the validation workshop in Sary Mogul, it was not apparent that Kyrgyz communities had historically used ecological calendars. However, during the validation workshop it became clear the cosmological relationships were embedded and informed by their habitat. This aspect of a universally shared practice was simultaneously accompanied by biophysical indicators that are unique to the cosmology of this particular agropastoral culture conveying their ecological and cultural distinctiveness (Fielstrup, 2002: 210–217; Schuyler, 1877: 329). The validation workshop firmly corroborated the shared legacy of ecological calendars across diverse ethnicities, ecological professions, and geographical locations.

**Fig. 4:**
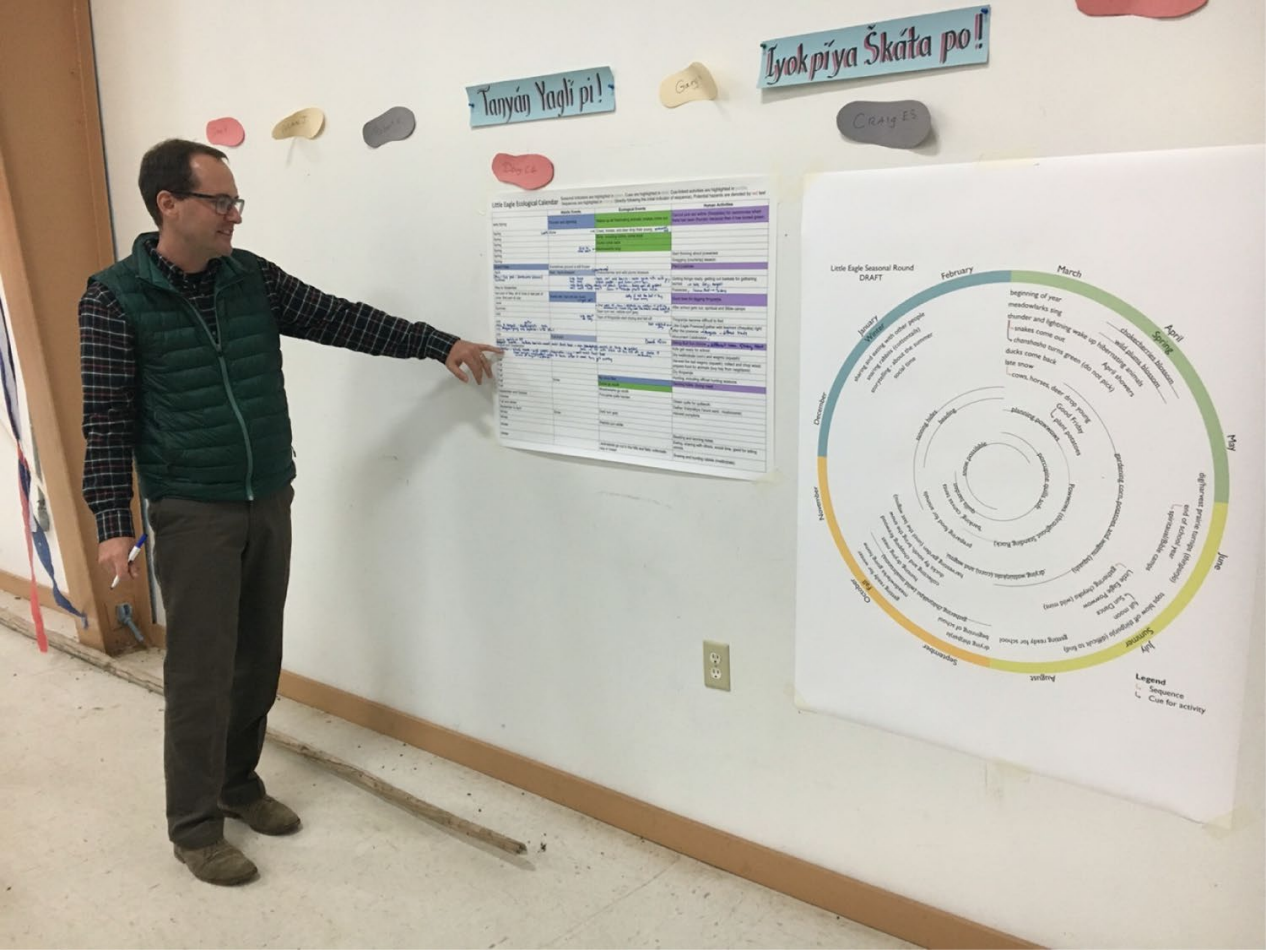
Dr. Morgan Ruelle undertaking validation of seasonal rounds at Standing Rock Sioux Nation, 2018. Photo Credit: Karim-Aly Kassam

Validation at Standing Rock Sioux Nation was conducted in six communities in October 2018 after similar workshops had been organized in the Pamirs. As a result, the research team had identified effective techniques that could be modified for these Native American communities to facilitate greater communication. For example, data collected during the first phase were presented in two forms: first, a seasonal round in table format as in the Pamirs; and second, a circular diagram in which the same information had been digitized (Fig. 4). As at the other sites, these validation activities were extremely important in that they revealed shortcomings of previous data and elicited more knowledge, in part due to participation of different individuals, and because participants could more easily envision the ultimate value of the research. As in Pamir communities, celestial indicators, such as the sun and moon are utilized by communities not as fixed occurrences in time but in direct relation to biophysical events such as “the moon when the cherries are black” or “the moon of snow blindness.”

At the validation workshop in the Oneida Lake watershed, information was presented as both a table and a circular diagram to facilitate discussion, correction, and new insights. The validation workshop was different in character and content than the project inception workshop. At Oneida Lake, the majority of the Euro-American settler community did not accept the existence of anthropogenic climate change on ideological grounds. However, in the validation workshop, discussion of climate change was much more nuanced, and the ideological divide was not a significant factor; collaboration and adaptation were seen as the priority. Furthermore, the conversation conveyed a greater sense of urgency. Keeping in mind that the project was initiated in 2016 during an unusually dry season where farmers had to cull their herds due to a lack of fodder; two and half years later, there was a palpable anxiety associated with livelihood activities being affected by unusual weather events. Moreover, valuable insights regarding the relationship between the fixed photoperiod and changing temperatures and precipitation were discussed in terms of impact on fish, plants, and poultry. Participants not only represented their own ecological professions but also represented various community associations, taking a long-term view and showing a concern for future generations. As in Standing Rock, there was a deep concern about loss of local ecological knowledge among the youth related to beekeeping, farming, fishing, herding, hunting, and orcharding. The dignity, mutual respect, mindfulness and care demonstrated by the participants at the validation workshops in the Oneida Lake watershed reflected similar qualities found among Elders in Indigenous communities in Central Asia and Standing Rock.

As an iterative process, the seasonal round facilitates validation at vital stages in the transdisciplinary process. It also provides an opportunity to identify areas of difference and synthesis which are essential to any knowledge cogeneration process. First, the validation process is about testing the credibility of the co-generated knowledge; specifically identifying seasonal indicators for developing anticipatory capacity to anthropogenic climate change. Second, it is also an exercise in communication by illustrating the value of each of the various knowledge systems and combining the diversity of expertise found within the community of enquirers as well as community of practice. Unlike the blind peer review process, in this form of validation, difference is valorized as community members and researchers engage in discussion to establish mutual understanding. Participants can see and respond to each other, using verbal as well as non-verbal communication. The process is transparent and shared, hence engendering dialog and reflection.

Because the research team is visiting the community of practice, there is opportunity for further collaborative reflection and new insights may emerge even after the formal validation meeting has concluded. In fact, after the validation there were opportunities for additional interviews and data collection related to specific biophysical indicators. Whereas in each of our study sites, one validation workshop has been conducted, it can be possible that several validation stages are needed or that further development of certain details have to be pursued.

Final validation workshops presenting a draft *ecological calendar* for each of the research sites were scheduled for 2020 but are delayed due to the COVID-19 pandemic. These validation workshops will present drafts of ecological calendars for use and implementation by each community. The *ecological calendar* will be framed by the transdisciplinary understanding gained by the co-generative process described above. Each *ecological calendar* will be simultaneously particular and universal. It will be unique as framed within the cosmology and ecological context of each research site and common in that it will be conveyed in a vocabulary and methodology familiar to all the communities so that they may be able to share insights with each other.

### Vista to Specific Disciplinary Research

The relationality revealed by articulating the seasonal round of each research site enables the members of the community of enquiry to (1) find elements within the rich information provided that was directly relevant to their respective discipline, and (2) to recognize the research pathway, value, and role of each contributing element from the varied ways of knowing assembled to the overall research aim. The process of generating the seasonal round simultaneously spurs humility by illustrating what is not known as well as generates mutual respect by showing the contributions other ways of knowing can make to one’s own discipline and understanding. This sets the stage for further transdisciplinary research feeding into the process of developing ecological calendars:

- Ethnographic interviews were undertaken to gather biophysical indicators, cues, and sequences of seasonal livelihood activities.
- Weather stations and soil loggers were installed in the mountain communities to measure key meteorological variables, as no long-term data records exist.
- Vegetation cameras were installed to monitor intra-annual changes in phenology and snow cover.
- Information on the vegetative and reproductive development of key agricultural species, as well as planting and harvesting times, was recorded with the assistance of farmers to use for phenological forecast modelling based on climate conditions.
- Farmers and herders were asked to keep diaries and take regular photographs to document phenology and weather conditions.
- Long-term regional climate data were downscaled and complemented with community observations (Haag *et al.*, 2021).

Having established the collaborative process, while walking through the village, people came out to tell stories, and wanted to show their gardens, greenhouses and other agricultural operations. This strong engagement of community members can provide further insights and inform new research ideas.

### Insight into Seasonal Change

Any strategy to anticipate seasonal weather dynamics and adapt to climate change needs to be grounded in the local sociocultural and ecological context. Climate models can capture regional weather processes but lack seasonal deterministic forecasting capacity at the scale of a village or valley, particularly in complex topographies (Hall, 2014; Kassam *et al.*, 2018). During the validation process in the three Pamir communities, climate scientists were invited to present results of a regional climate analysis to initiate a two-way learning process and to start discussions about weather processes at the local scale (Haag *et al.*, 2019). By sharing those results, communities can provide new insights on how local climate and weather conditions differ from the regional level. Those insights are particularly valuable, as local-scale climate records are scarce across the Pamirs and regional data sets cannot accurately capture small-scale variations of temperature and precipitation in complex terrain. For example, residents of Roshorv confirmed that they perceive a warming trend in spring, autumn and winter, but they disagreed with regional climate model data that summer is warming. In Savnob, further insights about changes in the timing of snow were shared, and in Sary Mogul community members disagreed that seasons are warming at all, except for winter. Building anticipatory capacity requires an understanding of local weather patterns and microclimatic conditions over the long-term. Microclimates are often oversimplified in large scale studies and gaining insight into their impact can be difficult and time consuming (Potter *et al.*, 2013). From the standpoint of communities of enquiry, a genuinely transdisciplinary methodology is necessary to engage the communities of practice who hold such knowledge and co-generate new systems that enhance their abilities to anticipate change.

Seasonal rounds are context-specific and, therefore, serve as an entry point for understanding a community’s sociocultural and ecological relationality. When collected from heterogeneous community members such as farmers, fishers, herders, hunters, orchardists and so on, the insights are revealing to the community of enquiry as well as to the community of practice. For the enquirers, the process yields theoretical insights about the relationship between climate data and local ecosystems, such as phenological indicators that maintain synchronies with seasonal change. For community members, a focused discussion of the seasonal round provides an opportunity to synthesize diverse knowledge about the seasonal rhythms of their habitat emerging from different livelihood activities.

Although much of this work has been conducted with Indigenous communities, the Oneida Lake watershed provided an opportunity to engage a predominately Euro-American settler community. Some of the workshop participants and interviewees in Oneida Lake were skeptical about anthropogenic climate change; however, they were excited to engage in a discussion of their seasonal activities, and willing to talk about how changes in the weather have affected their own livelihood activities. The discussion of seasonal rounds created a space for engagement around common interests and concerns, avoiding assumptions based on political or ideological positions or affiliations. In a telling moment, the lead principal investigator asked a participant, who is a hunter and tour operator: “What are your priorities and concerns?” After some hesitation, he answered: “I don’t know. I need time to think. We have never been asked about our needs. We are [usually] asked to take surveys.” After a long pause he began listing concerns that directly linked his seasonal livelihood activities to the ability to anticipate climatic variation. The process of making seasonal rounds facilitated genuine engagement. At this early stage in research, the primary role of the community of enquiry is to listen. A foundational element of using seasonal rounds is the understanding that knowledge does not emanate from the heads of experts but through a community of practice’s engagement with their habitat. Thus, creating a responsive space, to co-generate original, empirical insights about seasonal change.

The overall process of engagement makes explicit the seasonal round, which requires the participation of the community of practice, who have not observed it in its full scope with the heterogeneity of points of view and nuanced detail according to varied community members. The role of the community of enquiry in the co-creation is not only contributing diverse expertise but integrating knowledge with different insights provided by community members, tying it together, only to revisit it collectively and iteratively.

## Conclusion

This paper is about a methodology to understand the priorities of diverse communities in response to a global phenomenon, namely anthropogenic climate change, that is experienced uniquely in different cultural and ecological contexts. The community of enquiry is a learning agent. Its first and foremost task is to hear and then listen, as described in this methodology. Diverse researchers, including students, need to understand how communities of practice comprehend environmental change.

**Fig. 5:**
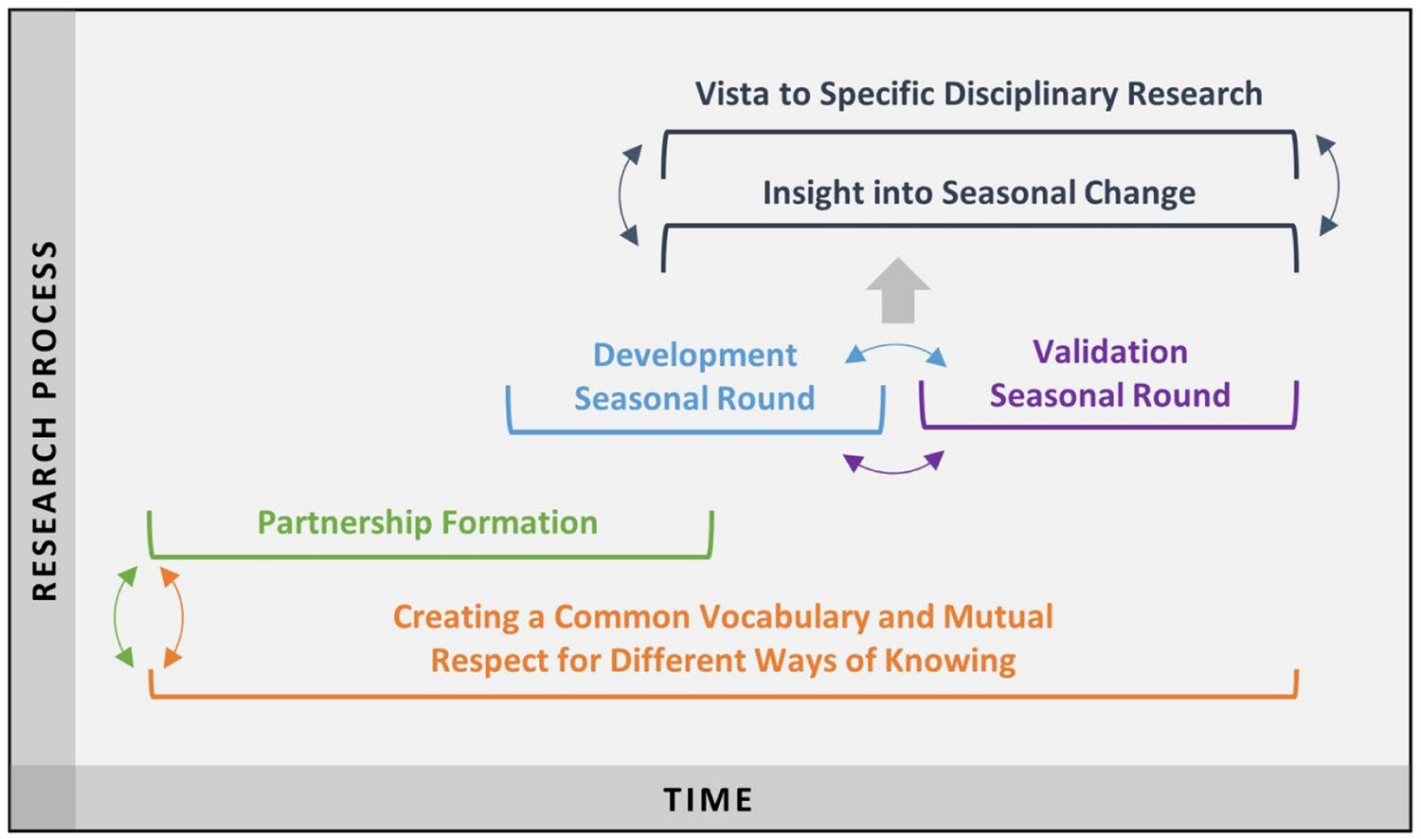
Temporal visualization of the transdisciplinary research process

Seasonal rounds are an articulation of a community’s engagement of the sociocultural with the ecological in their habitat. The historical overview of the evolution of seasonal rounds reveals that while these rounds were originally substantial and nuanced ethnographic descriptions of spatial and temporal expressions of an Indigenous people’s seasonal relationship with their ecological habitats, these relationships can be visualized in support of transdisciplinary research aimed at understanding climate change at the local level. The process of visualization facilitates communication across cultural and disciplinary boundaries. A temporal visualization of the research process reveals that a respectful partnership between the community of practice and the community of enquiry allows development of a common vocabulary that is foundational to an emergent understanding of seasonal change and specific disciplinary insights. The development of the seasonal round and the iterative process of validation then contribute to co-generated knowledge that is transdisciplinary in character (Fig. 5). This co-generated knowledge is a key building block to developing anticipatory capacity for climate change using ecological calendars.

A key feature of this process was the challenge of time as relational compared to fixed time as in the Gregorian calendar, which is a legacy of colonialization. Both the communities of practice and enquiry struggled with these conceptions of time. However, resolution of one or the other was not the objective because these conceptions of time coexist, mutually affecting people’s livelihoods and mindsets. Our research objective was to create an enabling environment where time as relational is also considered because it is grounded in the history and consciousness of both the communities of enquiry and practice. This was not an epistemological challenge but an ontological one as it spoke to a community’s livelihood and way of being. Both conceptions of time were present when co-generating insight by interpreting empirical observations. The response to climate change will demand exploring pluralistic conceptions of time rather than relying on narrower and more recent mechanistic conceptions arising from industrialization.

Seasonal rounds are simultaneously particular or context-specific to individual Indigenous or rural societies across the planet as well as universal in terms of a shared human heritage and practice of application of this spatial and temporal knowledge transmitted across generations over several millennia. Over these millennia, climatic variation has transformed habitats and altered use of resources within them, thereby affecting peoples’ food and livelihood systems.

In order to make explicit the value of this ethnographic approach to understanding and building adaptive capacity to anthropogenic climate change, we recognize two aspects of the spatial and temporal dimensions of seasonal rounds. The spatial feature addresses seasonally determined *movement* and *occupancy* across landscapes. The temporal factor provides insights into *seasonal indicators* and *cues* while simultaneously building a cumulative body of knowledge within a community to *transfer to future generations*. Because human knowledge derives from engagement and performance in the fluidity of spatial and temporal dimensions of climatic and ecological cycles, co-generated articulation of seasonal rounds facilitates adaptation to the evolving reality of anthropogenic climate change. The process generates empirically rich Indigenous or local knowledge which has the potential to inform gaps in the biological, physical and social sciences. Acknowledging differences among and within the community of enquiry and community of practice is not a weakness. Effective partnership between the community of practice and the community of enquiry requires combining knowledge obtained from different spatial and temporal scales and ontologies. As the rapidity of anthropogenic climate change is breaking the linkages that are foundational to this spatial and temporal understanding, this methodological approach provides an enabling environment for co-creation of knowledge that may help anticipate climatic variation for Indigenous and rural societies through effective adaptation strategies.

## Supplementary Information

Below is the link to the electronic supplementary material.

Supplementary file1 (DOC 32 KB)

Supplementary file2 (DOC 37 KB)

## Footnotes

1 We are aware of the subtle epistemological and historical differences between Indigenous and local forms of knowledge, including conceptions of time (Hatfield *et al.*, 2018). Our aim is cognitive diversity arising from placed-based (Indigenous and local) understanding. Unlike local knowledge, when we make reference to “Indigenous” ways of knowing, we recognize their autochthonous character through complex connectivity to their habitat. “Indigeneity” is more than the earliest known inhabitant; rather it identifies a self-consciousness of being descended from ancestors who were born out of the earth and how this consciousness continues to guide daily actions, a way of being, or ontology of a community (Kassam *et al.*, 2018: 98).

2 By anticipatory capacity we mean the ability of these societies to envision and enact a desirable future for their livelihoods and food systems.

3 We use “revitalization” to acknowledge the decolonization of indigenous communities and their knowledge systems that have undergone oppression and cultural erasure, if not outright genocide.

4 We use the terms “cogeneration” or “cocreation” rather than “coproduction” to indicate an organic process with human agency, since production references an industrialized and mechanized conception of knowledge common to the command and market economies.

5 Boas marveled at the geographic knowledge the Inuit communities had of their habitat which fundamentally informed his own map-making agenda (Kassam, 2009).
