# Supplementary material for: Engaging Transformation: Using Seasonal Rounds to Anticipate Climate Change
Source: Hum Ecol Interdiscip J. 2021 Sep 29;49(5):509–23. doi: 10.1007/s10745-021-00269-2 (PMC8479261; doi:10.1007/s10745-021-00269-2)
Supplement: Supplementary file 1 — Supplementary file1 (DOC 32 KB) [file 10745_2021_269_MOESM1_ESM.doc]

**Engaging Transformation: Using Seasonal Rounds to Anticipate Climate Change**

**Supplementary Material 1**

**Past Use of Seasonal Rounds in Participatory Research**

Since the 1990s, seasonal rounds have been used to gain insight into the depth and breadth of a people’s relations with their habitat. Brief summaries of these projects demonstrate the diverse cultural contexts in which seasonal rounds have been applied to a wide range of community-driven projects:

- Robinson and Kassam (1998) documented the seasonal rounds of the Sami on the Kola Peninsula in Lovozero (Russia) to facilitate recognition of indigenous land-use rights after the collapse of the Soviet Union. The spatial movement of Sami reindeer herds through the seasons provided context-specific understanding of relations with other ungulates such as elk and wild reindeer (caribou), fur bearing animals, fish, waterfowl, raptors, trees, medicinal plants and berries across taiga and the tundra.
- Kassam and Soaring Eagle Friendship Center (2001) led a participatory research project with Dene women of Hay River, Northwest Territories, Canada. The articulation of seasonal rounds yielded information on their historic and current connectivity to the boreal forest. Specifically, the women’s and their kinfolk’s relationship with their habitat including hunting, fishing, trapping of specific animals; gathering of medicinal plants and berries; and spiritual engagement with features of the northern landscape illustrated their past and continued land use despite a tragic history of colonization and subsequent devastation caused by disease and sedentarization.
- Kassam (2009; with the Wainwright Traditional Council 2001) conducted a transdisciplinary applied research project on the impact of chemical pollutants on the food systems of Arctic coastal communities, including documentation of seasonal rounds of the Iñupiat community of Wainwright, Alaska, USA and the Inuvialuit community of Ulukhaktok (Holman), Northwest Territories. Seasonal rounds provided information on species of marine and terrestrial mammals, fish, birds, and plants that were part of their food system. Documenting these communities’ human ecological relations in time and space has meant that oil and gas development initiatives have to be cognizant of the complex connectivity of Indigenous peoples. Illustrating seasonal rounds highlighted the significance and vulnerability of biodiversity within the local food system.
- Ruelle and Kassam (2011, 2013) conducted research with Elders in the Standing Rock Nation of North and South Dakota (USA) to understand how revitalization of relations with plants used in traditional foodways might address diet-related disease. Given the diverse experiences of Elders across heterogeneous environments, use of plants for different purposes, and strategies for gathering multiple species, each Elder illustrated her or his own seasonal round, which were overlaid with high transparency to reveal patterns of convergence and differentiation. The compiled round was used to plan activities for youth and elders to gather plants together and strengthen their knowledge of plants.
- Ruelle (2015; et al. 2019) documented how subsistence farmers in the Semien Mountains of Ethiopia use hundreds of domesticated and non-domesticated plants species in their food system. Increasing unpredictability in patterns of precipitation require strategic timing of planting and harvesting field crops. Discussion and analysis of the seasonal round revealed important synchronies between domesticated and non-domesticated species that could serve as effective seasonal cues for agriculture.

**References**

Kassam, K.-A. S. (2009). *Biocultural Diversity and Indigenous Ways of Knowing: Human ecology in the Arctic*. Calgary: University of Calgary Press.

Kassam, K.-A. S., & Soaring Eagle Friendship Center. (2001). *So That Our Voices Are Heard: Forest Use and Changing Gender Roles of Dene Women in Hay River, Northwest Territories*. Calgary: CIDA-Shastri Partnership Programme.

Kassam, K.-A. S., & The Wainwright Traditional Council. (2001). *Passing on the Knowledge: Mapping Human Ecology in Wainwright, Alaska*. Calgary: Arctic Institute of North America.

Robinson, M., & Kassam, K. S. (1998). *Sami Potatoes: Living with Reindeer and Perestroika*. Calgary: Bayeux Arts.

Ruelle, M. L. (2015). *Human-plant ecology of an Afromontane agricultural landscape: Diversity, knowledge, and food sovereignty in Debark, northern Ethiopia*. Cornell University, Ithaca, New York.

Ruelle, M. L., & Kassam, K.-A. S. (2011). Diversity of plant knowledge as an adaptive asset: A case study with Standing Rock Elders. *Economic Botany*, *65*(3), 295–307. doi:10.1007/s12231-011-9168-x

Ruelle, M. L., & Kassam, K.-A. S. (2013). Foodways transmission in the Standing Rock Nation. *Food and Foodways*, *21*(4), 315–339. doi:10.1080/07409710.2013.850007

Ruelle, M. L., Kassam, K.-A. S., Morreale, S. J., Asfaw, Z., Power, A. G., & Fahey, T. J. (2019). Biocultural diversity and food sovereignty: a case study of human-plant relations in northwestern Ethiopia. *Food Security*, *11*(1), 183–199. doi:10.1007/s12571-019-00888-0
