# Supplementary material for: Engaging Transformation: Using Seasonal Rounds to Anticipate Climate Change
Source: Hum Ecol Interdiscip J. 2021 Sep 29;49(5):509–23. doi: 10.1007/s10745-021-00269-2 (PMC8479261; doi:10.1007/s10745-021-00269-2)
Supplement: Supplementary file 2 — Supplementary file2 (DOC 37 KB) [file 10745_2021_269_MOESM2_ESM.doc]

**Engaging Transformation: Using Seasonal Rounds to Anticipate Climate Change**

# Supplementary Material 2

# Brief Descriptions of Collaborative Research Sites

Below is a description of each of the locations for participatory research involving seasonal rounds.

## Standing Rock Sioux Nation, USA

The Standing Rock Nation is located in the Northern Great Plains of the United States, encompassing 9300 km^2^ west of the Missouri River in North and South Dakota. The population of Standing Rock (8,581 as of 2018) is predominately Native American (78.2%), mainly Lakota and Dakota. Most of the reservation’s forests were lost following the construction of the Oahe Dam by the U.S. Army Corps of Engineers in the 1950s (Ruelle, 2017). Beginning in 2016, Standing Rock was the site of international protests against the construction of the Dakota Access Pipeline across the Missouri at the northern edge of the reservation. The seat of tribal government is located at Fort Yates. Seasonal rounds were developed with eight communities, including Fort Yates, Cannonball, Solen, Porcupine, Kenel, Little Eagle, Bullhead, and Wakpala.

## Oneida Lake, New York, USA

Oneida Lake, a remnant glacier lake, is located in central New York State, to the east of the city of Syracuse and south of Lake Ontario. It is a shallow lake with an average depth of 6.8 m covering around 207 km^2^ (Rudstam et al., 2016). The Oneida Lake Watershed includes 3,877 km^2^, which is home to 1,151 farms. The average farm size is approximately 78 hectares (USDA, 2017).

The research included communities living throughout the Oneida Lake Watershed. As a Euro-American settler community, a significant portion are working as farmers and in other ecological professions (e.g., hunting, trapping, fishing, and more); half of interviewees have engaged these professions for more than 20 years, and many had parents or grandparents following the same professions in the region.

## Sary Mogul, Alai Valley, Kyrgyzstan

Sary-Mogol is part of the Alai Region of Osh Oblast (province) of Kyrgyzstan. Ranging from 2900 - 3100 meters a.s.l., the village is situated in the Alai Valley of southern Kyrgyzstan along the northern margin of the Pamir Mountains. The majority of inhabitants are of Kyrgyz descent, practicing Sunni Islam. The Kyrgyz language belongs to the Altaic family and has different origins than their Pamiri neighbors. The total population of the village is 5165 people (Ayil Ökmötü Sary-Mogol, 2010). There is a long history of agropastoral activities in the valley (Bernshtam, 1950 p. 187-188), which remains the basis of the economy (Shirasaka et al., 2016). Although, many families are still involved in seasonal livestock keeping and small-scale cropping, people also work in local institutions like shops, in the bank, or at the coal mine.

## Savnob, Bartang Valley, Tajikistan

The village of Savnob is located in the Bartang Valley in the autonomous district of Gorno-Badakhshan (GBAO) in the Pamir Mountains of Tajikistan. The Bartang Valley is a nexus of diversity because the Silk Road provided contact to a variety of other cultures. Savnob is situated on a narrow terrace bounded on one side by the Bartang Gorge and on the other by a steep rocky slope. The 310 inhabitants occupy only 10 ha arable land. Belonging to the Shia Ismaili faith, they speak an Indo-European language of the Pamiri family. Although most interviews were conducted with individuals who professionally identified as farmers and teachers, rather than herders, it is common for many people to have additional professions. Often male community members migrate to Russia to send remittances for support. This migratory movement while positive in terms of potential income, has significant implications for families, the gender burden of labor upon women, and transmission of agricultural knowledge. Communities in the Bartang Valley are particularly vulnerable to climatic variation because they are located at high elevations. Furthermore, they have withstood the collapse of the Soviet command economy followed by nearly a decade-long civil war. In 2006, while undertaking field work in Savnob, we were first introduced to calendar of the human body, a context-specific ecological calendar (Kassam et al., 2011). Therefore, this village provided the impetus for our current research.

## Roshorv, Bartang Valley, Tajikistan

Roshorv is also located in the Bartang Valley of the GBAO in the Pamir Mountains of Tajikistan and has withstood similar pressures and challenges as Savnob. The village is situated on a gently sloping plateau and a glacier-fed stream supports its agriculture. This geographical setting provides for a larger area of arable land (150 ha) than in Savnob, supporting a greater population of 1,200 inhabitants, most of whom are subsistence farmers and livestock herders.

## Baharak and Jurum, Badakhshan, Afghanistan

The original site for our collaborative research was at Lake Shiva in the Shugnan valley of Badakhshan, Afghanistan. However, this region was overrun by invading extremists. Therefore, in 2018, the districts of Baharak and Jurum were chosen. Baharak, situated on a spacious alluvial fan in the Kokcha valley, is surrounded by intensively used arable land. Jurum is located along a tributary of the Kokcha river. Baharak and Jurum, while sharing a close cultural, linguistic and religious connections with the Pamiri communities of Tajikistan, are mostly Sunni Muslims. Research in this area is precarious because of Taliban presence.

**References:**

Ayil Ökmötü Sary-Mogol, 2010. Letter from the municipal administration to the responsible administration of the Osh Oblast concerning boundary change, including land use statistics and historical information. Sary-Mogol, Kyrgyzstan.

Bernshtam, A.N., 1950. Istoriko-arkheologicheskie ocherki tsentral’nogo Tian’-Shania i Pamiro-Alaia. Izd-vo Akademii nauk SSSR, Moscow.

Kassam, K.-A.S., Bulbulshoev, U., Ruelle, M.L., 2011. Ecology of time: Calendar of the human body in the Pamir Mountains. J. Persianate Stud. 4, 146–170.

Rudstam, L.G., Jackson, J.R., Hetherington, A.L., 2016. Concluding remarks: Forecasting the future of Oneida Lake and its fishery in an era of climate change and biological invasions., in: Rudstam, L., Mills, E., Jackson, J., Stewart, D. (Eds.), Oneida Lake: Long-Term Dynamics of a Managed Ecosystem and Its Fishery.

Ruelle, M.L., 2017. Ecological relations and Indigenous food sovereignty in Standing Rock. Am. Indian Cult. Res. J. 41, 113–125. doi:10.17953/aicrj.41.3.ruelle

Shirasaka, S., Song, F., Watanabe, Teiji, 2016. Diversity of seasonal migration of livestock in the Eastern Alai Valley, Southern Kyrgyzstan, in: Kreutzmann, H., Watanabe, T. (Eds.), Mapping Transition in the Pamirs. pp. 127–143. doi:10.1007/978-3-319-23198-3_9

USDA, 2017. Census of Agriculture: County Data [WWW Document]. United States Dep. Agric. URL www.nass.usda.gov/Publications/AgCensus/2017
